# Pragmatic skills predict online counterfactual comprehension: Evidence from the N400

**DOI:** 10.3758/s13415-016-0433-4

**Published:** 2016-05-09

**Authors:** Eugenia Kulakova, Mante S. Nieuwland

**Affiliations:** 1 Centre for Cognitive Neuroscience, Department of Psychology, University of Salzburg, Salzburg, Austria; 2 Department of Psychology, School of Philosophy, Psychology and Language Sciences, University of Edinburgh, Edinburgh, UK

**Keywords:** Counterfactuals, Pragmatics, Event-related potentials, N400, Autistic traits

## Abstract

Counterfactual thought allows people to consider alternative worlds they know to be false. Communicating these thoughts through language poses a social-communicative challenge because listeners typically expect a speaker to produce true utterances, but counterfactuals per definition convey information that is false. Listeners must therefore incorporate overt linguistic cues (subjunctive mood, such as in *If I loved you then*) in a rapid way to infer the intended counterfactual meaning. The present EEG study focused on the comprehension of such counterfactual antecedents and investigated if pragmatic ability—the ability to apply knowledge of the social-communicative use of language in daily life—predicts the online generation of counterfactual worlds. This yielded two novel findings: (1) Words that are consistent with factual knowledge incur a semantic processing cost, as reflected in larger N400 amplitude, in counterfactual antecedents compared to hypothetical antecedents (*If sweets were/are made of sugar*). We take this to suggest that counterfactuality is quickly incorporated during language comprehension and reduces online expectations based on factual knowledge. (2) Individual scores on the Autism Quotient Communication subscale modulated this effect, suggesting that individuals who are better at understanding the communicative intentions of other people are more likely to reduce knowledge-based expectations in counterfactuals. These results are the first demonstration of the real-time pragmatic processes involved in creating possible worlds.

**Electronic supplementary material:**

The online version of this article (doi:10.3758/s13415-016-0433-4) contains supplementary material, which is available to authorized users.

Counterfactual “what-if” thought enables people to go beyond the here and now and into possible and impossible worlds. This ability to imagine alternative realities plays an important role in cognition and emotion (Byrne, 2016; Rips & Edwards, 2013; Spellman & Mandel, 1999). Thinking about alternative sequences of events helps people to infer causality and adapt their future behaviour accordingly. Experiencing counterfactual emotions, such as regret or relief, contributes to an understanding of personal control and responsibility (Epstude & Roese, 2008; Frith, 2013). Although humans routinely and spontaneously engage in counterfactual thought, communicating these thoughts via language poses a social-communicative challenge: Human communication is effective because speakers typically say things that are true (Grice, 1975), but counterfactuals per definition convey information that is false. Counterfactual antecedents therefore contain overt linguistic cues (e.g., *If I loved you then*) that guide listeners towards the intended meaning. Language comprehension requires rapid incorporation of such cues into the unfolding interpretation in order to avoid misunderstanding. This implies an important link between social cognition and online counterfactual language comprehension. To investigate this link, the current study used event-related brain potentials (ERP) to test whether the pragmatic ability to interpret others’ communicative intentions impacts the online comprehension of counterfactual antecedents.

## Social cognition and counterfactual language

Understanding the intentions of other people is a crucial element of social cognition and involves the attribution of mental states known as theory of mind (ToM; Baron-Cohen, Leslie, & Frith, 1985; Premack & Woodruff, 1978). In clinical and developmental research, ToM reasoning has long been associated with counterfactual thought (Scott, Baron‐Cohen, & Leslie, 1999; Perner, Sprung, & Steinkogler, 2004), perhaps because ToM and counterfactuals both involve two representations of incompatible information (two different perspectives in ToM reasoning, false and true in counterfactual reasoning). Developmental studies show that children who are better at counterfactual reasoning are also better at the false belief task, a standard measure of ToM capacity (Peterson & Bowler, 2000; Riggs, Peterson, Robinson, & Mitchell, 1998; Wimmer & Perner, 1983). Neuroimaging studies with adult participants show that counterfactual reasoning and ToM reasoning draw upon overlapping brain regions (Van Hoeck et al., 2014). Moreover, children with autism, a developmental disorder characterized by both pragmatic and ToM deficits (Baron-Cohen et al., 1985), exhibit impairments in counterfactual reasoning (Grant, Riggs, & Boucher, 2004; Leevers & Harris, 2000) despite performing well on other types of reasoning tasks (McKenzie, Evans, & Handley, 2010; Scott & Baron-Cohen, 1996). Impaired counterfactual reasoning in children with autism may therefore reflect a pragmatic deficit. Just as children with autism miss the social purpose of pretence play (Leslie, 1992), they may not understand the communicative intention to temporarily accept a false proposition as true in order to reason about it (e.g., Leevers & Harris, 2000; Surian, Baron-Cohen, & Van der Lely, 1996; see also Baron-Cohen, 2008). Such pragmatic deficit could impede the successful set-up of a counterfactual possible world (*If elephants had wings…*), subsequently interfering with counterfactual reasoning (… *then they could fly*). Although the exact relationship between pragmatic skills and ToM is not clear (Cummings, 2013; Tager-Flusberg, 2000), these abilities show robust positive correlations and are possibly supported by a common neural system (Martin & McDonald, 2003). Taken together, developmental, clinical, and neuroimaging findings suggest a close relationship between pragmatic skills and counterfactual reasoning.

This relationship may have repercussions for online counterfactual language comprehension. Because literal and overt meaning vastly underdetermines speaker’s meaning (Sperber & Wilson, 2002), understanding the meaning of what other people say heavily relies on inferences about people’s intentions. These pragmatic inferences are based on the cooperative principles of communication (Levinson, 1983; Van Linden & Verstraete, 2008) and must be generated in a rapid and incremental manner to avoid delays in comprehension. The most important cooperative principle is to be truthful, to say what you mean and not say what you do not mean (Grice, 1975). Counterfactuals are pragmatically challenging because they also convey information that is factually false. Counterfactual comprehension therefore critically relies on successful comprehension of the subjunctive mood (e.g., *If Mary had tossed tails, she would have won*). Subjunctive mood is the linguistic cue that the speaker knows that what he or she is saying is factually not true (Stalnaker, 1975) and expects the hearer to know the same. Subjunctive mood can be contrasted with indicative mood (*If Mary tossed tails, she won*), which is used to convey purely hypothetical conditional relations and does not restrict the truth-value of the expressed proposition (Byrne, 2002; Stewart, Haigh, & Kidd, 2009).

In the current study, we tested the hypothesis that pragmatic skills impact the online comprehension of counterfactual antecedents. We contrasted comprehension of subjunctive and indicative mood, which use the same *If*-construction and therefore allows for a well-matched investigation of counterfactuality (Kulakova, Aichhorn, Schurz, Kronbichler, & Perner, 2013). Our participants read counterfactual or hypothetical antecedents, such as *If sweets were/are made out of sugar*, while their electrical brain activity was measured. Following recent ERP studies on pragmatic language comprehension (Ferguson, Cane, Douchkov, & Wright, 2015; Ferguson & Cane, 2015; Nieuwland, Ditman, & Kuperberg, 2010; Nieuwland & Kuperberg, 2008; Van den Brink et al., 2012), our dependent measure was the N400 ERP (Kutas & Hillyard, 1980), an ERP component that reflects the impact of online semantic expectations during language comprehension. Before outlining our ERP predictions, we discuss the rationale of our study.

## The N400 ERP and online pragmatic comprehension

Our dependent measure, the N400, is a negative-going deflection that peaks over centro-parietal electrodes around 400 ms after word onset (Kutas & Hillyard, 1980) and that is elicited by every content word of an unfolding sentence. N400 amplitude decreases when retrieval of word-associated information in semantic memory is facilitated by the context, potentially via knowledge-based predictions (e.g., Ito, Corley, Pickering, Martin, & Nieuwland, 2016; Kutas & Federmeier, 2011). Such predictions are also reflected in ‘sentence truth-value N400-effects’, when words that render a sentence true and plausible elicit a smaller N400 than words that render a sentence false or implausible (Nieuwland, 2015, 2016; Nieuwland & Kuperberg, 2008). Such effects seem not to directly reflect the online computation on truth-value, but to reflect people’s use of real-world knowledge as well as pragmatic knowledge to generate expectancies about upcoming words. This follows from observations that N400 amplitude is not a direct function of propositional plausibility or truth-value, but instead a function to what extent the incoming word shares semantic features with information that people may be expecting to appear (e.g., Ito et al., 2016; Kutas & Federmeier, 2011). When an incoming word is consistent with these knowledge-based predictions, the semantic retrieval of relevant information is facilitated, leading to smaller N400s compared to words that are inconsistent with world knowledge (Hagoort, Hald, Bastiaansen, & Petersson, 2004; Nieuwland, 2013, 2015, 2016; Nieuwland & Kuperberg, 2008; Nieuwland & Martin, 2012).

Context-based facilitation of semantic processing can occur regardless of whether the described context is factual or counterfactual (Ferguson, Scheepers, & Sanford, 2010; Ferguson & Cane, 2015; Nieuwland, 2013; Nieuwland & Martin, 2012). Furthermore, facilitating contextual information is not restricted to the literal meaning of words. Pragmatic cues such as communicative conventions or social-context information can be rapidly incorporated in the interpretation of the unfolding message, facilitating consistent continuations (Nieuwland et al., 2010; Nieuwland & Kuperberg, 2008). However, such pragmatic facilitation may depend on participants’ individual sensitivity to or use of available pragmatic cues. For instance, in an ERP study on comprehension of *some*-sentences, the N400 increase for underinformative sentences, such as *Some people have lungs*, was positively correlated with pragmatic skills (Nieuwland et al., 2010). Similar effects have been observed for individual differences in empathy, a ToM component engaged in identifying and responding to others’ emotions (Baron-Cohen & Wheelwright, 2004). Van den Brink et al. (2012) found that higher empathy quotient scores are associated with larger N400s in response to inconsistencies between message content and speaker identity, suggesting that empathy was associated with knowledge-based predictions from pragmatic cues, in this case, the speaker’s voice. In a recent study (Ferguson et al., 2015), empathy quotient scores were also associated with false belief reasoning during story comprehension, evidenced by larger N400s to words that suggested that a protagonist acted on information they could not possess. Taken together, the available ERP studies on pragmatic comprehension suggest that individual differences in pragmatic abilities can predict people’s use of pragmatic knowledge to generate expectations about upcoming information.

In ERP research on counterfactual language comprehension, all studies so far have examined the impact of a counterfactual antecedent on the comprehension of a factually false consequent (Ferguson & Cane, 2015; Ferguson, Sanford, & Leuthold, 2008; Kulakova, Freunberger, & Roehm, 2014; Nieuwland 2013; Nieuwland & Martin, 2012; Urrutia, de Vega, & Bastiaansen, 2012). While such a manipulation taps into important component of conditional reasoning, it does not capture the most characteristic feature of counterfactuality: the creation of the *if*-antecedent that is factually false but temporarily accepted as true. This process of creating counterfactual worlds remains poorly studied despite being a fundamental step towards conditional reasoning. It requires the rapid incorporation of subjunctive mood so that the comprehender can adjust his or her knowledge-based expectations. By marking the antecedent as counterfactual, subjunctive mood facilitates counterfactual continuations, possibly by lowering expectations of factually true continuations (Stewart et al., 2009; for a review, see Kulakova & Nieuwland, 2016). This lowering of expectations should lead to a larger N400 for a word that is consistent with real-world knowledge while not increasing the expectation of words that are both false and unrelated to the context (for a discussion, see Nieuwland, 2016). Moreover, if pragmatic skills determine the successful incorporation of subjunctive mood, pragmatic skills should correlate with comprehenders’ readiness to lower their expectations from real-world knowledge.

## The present study

In the present study, we tested whether pragmatically skilled participants were more likely to use the pragmatic cue of subjunctive mood in the counterfactual antecedent in order to set up a counterfactual interpretation. We recorded participants’ EEG while they read counterfactual and hypothetical conditional sentences with antecedents that conveyed information that was either false or true with respect to factual world knowledge.^1^ Counterfactual false antecedents (*If words were made out of sugar*) were phrased in subjunctive mood and expressed a state of affairs which is false with respect to world knowledge. In contrast, counterfactual true conditions (*If sweets were made out of sugar*) conveyed factually true knowledge and therefore violated the pragmatic cue of counterfactuality signalled by subjunctive mood. Hypothetical conditionals in both truth-value conditions were phrased in indicative mood (*If words/sweets are made out of sugar*) and served as a comparison condition with pragmatically unrestricted truth-values. Declarative sentences (*As words/sweets are made out of sugar*) were included as a nonconditional control condition. By measuring participant’s self-reported pragmatic skills (the Autism Quotient Communication subscale; Baron-Cohen, Wheelwright, Skinner, Martin, & Clubley, 2001), we investigated whether differences in social-communicative abilities can predict the generation of pragmatic expectations during conditional antecedent processing. We expected that participants would rapidly incorporate the pragmatic cue of counterfactuality and decrease the expectation of real-world consistent words, leading to larger N400s to counterfactual true antecedents compared to hypothetical true antecedents. In addition, we predicted that this counterfactual-pragmatic N400 effect would increase with the pragmatic skills of the participant. No effect of mood was expected for false antecedents, because counterfactual antecedents were not designed to increase the predictability of the factually false continuation (*If words were/are made out of sugar*).

## Method

### Participants

Thirty right-handed University of Edinburgh students (19 women, mean age = 22 years, *SD* = 4 years) gave written consent to participate in the study. All were native English speakers without neurological or psychiatric disorders and were paid for their participation. The study was conducted in accordance with the Declaration of Helsinki and was approved by the University of Edinburgh Ethics Committee.

### Materials and design

We constructed 90 sentences about various world-knowledge topics with which native English-speaking Edinburgh University students were expected to be familiar. Each sentence could be phrased as counterfactuals (subjunctive mood) or hypotheticals (indicative mood) and have an antecedent either true or false in respect to factual world knowledge (see Table 1 for example stimuli). In addition to these experimental conditions, stimuli could be phrased as factual declarative phrases. These were identical to the indicative conditionals except that they started with *As* instead of *If*. In both experimental and declarative conditions, the second word of the first clause manipulated real-world truth-value and could be either consistent (*sweets*) or inconsistent (*words*) with the critical word (*sugar*), resulting in factually true or false initial clauses, respectively. The critical words were identical across all conditions and always took the final position of the first clause, with the distance between critical word and the noun which manipulated truth-value kept constant with four words. The experimental antecedents were followed by consequents that completed the conditional in a relatively plausible manner (“*… sentences/candy can/could make people very fat when consumed frequently*). The complete stimulus list can be found in the Supplementary Material.

**Table 1:** Example stimuli of experimental conditional (counterfactual and hypothetical) and declarative control clauses

Condition	Example stimuli
Counterfactual-true	If sweets were made out of sugar,
	If light was perceived with the eyes,
	If flutes were used to make music,
Counterfactual-false	If words were made out of sugar,
	If sound was perceived with the eyes,
	If vegetables were used to make music,
Hypothetical-true	If sweets are made out of sugar,
	If light is perceived with the eyes,
	If flutes are used to make music,
Hypothetical-false	If words are made out of sugar,
	If sound is perceived with the eyes,
	If vegetables are used to make music,
Declarative-true	As sweets are made out of sugar,
	As light is perceived with the eyes,
	As flutes are used to make music,
Declarative-false	As words are made out of sugar,
	As sound is perceived with the eyes,
	As vegetables are used to make music,

*Note.* Critical words are underlined for expository purposes only.

Semantic similarity or lexical-semantic association values were calculated between critical antecedent-final words and the preceding clause using latent semantic analysis (Landauer, Foltz, & Laham, 1998). Mean LSA values in true conditions were significantly higher than in false conditions (0.25 (0.08) vs. 0.15 (0.11); *t*(89) = 9.12, *p* < .001).

It is important to note that the counterfactual antecedents were not designed to guide participants towards the expectation of one particular false continuation. To establish the effect of predictability of critical words, we collected cloze ratings from 81 student participants (59 females, mean age 19 years, *SD* = 2 years) using an online survey. Subjects completed one of six counterbalanced lists with the first clause of the sentences truncated before the critical word. Cloze value was computed as the percentage of participants who used the intended critical word (Taylor, 1953). In the experimental stimuli a 2 (truth-value: true, false) × 2 (mood: counterfactual, hypothetical) repeated-measures ANOVA (by items) showed that cloze values only differed between true (mean cloze value of counterfactual-true: .30; hypothetical-true: .31) and false (counterfactual-false: .04; hypothetical-false: .02) clauses, *F*(1, 89) = 125.75, *p* < .001, but not between mood, *F*(1, 89) < 1. In declarative sentences, continuations that rendered the first clause true were more predictable than false continuations (.41 vs. .01), *F*(1, 89) = 167.44, *p* < .001. However, it is possible that cloze rating participants ignored the factor of mood and used the first word that came to their mind regardless of whether it made counterfactual sense. We therefore report cloze values but do not refer to these values to explain the observed N400 effects. Importantly, if taken at face value, cloze values do not offer a simple alternative explanation to our online findings.

For the EEG experiment, six presentation lists were created. Each participant saw two conditions of each of the 90 stimuli but never in the same context that manipulated truth-value. Each subject therefore read 30 items per condition (four conditional-experimental conditions, two declarative control conditions). The sentences were pseudorandomized and interspersed with 64 plausible declarative filler sentences (e.g., *To mail a letter you have to put it in the postbox*). Each subject read a total of 244 sentences, which took approximately 50 minutes.

### Procedure

Participants silently read sentences from a monitor (black letters, light-grey background), presented word-by-word at a regular pace (300 ms word duration, 200 ms interword interval) with antecedent-final and sentence-final words presented for 600 ms. Antecedent-final critical words were presented with a comma and sentence-final words with a period. Each sentence was followed by a fixation cross upon which participants started the next sentence by button-press in a self-paced manner. Five practice trials at the beginning of the experiment familiarised participants with the procedure and presentation pace. There was no task except reading for comprehension. After the ERP experiment, participants filled out the Communication subscale of the Autism Spectrum Questionnaire (AQ-Comm; Baron-Cohen et al., 2001), which as been employed in previous ERP investigations of pragmatic language processing (Nieuwland et al., 2010). The scale comprises 10 items that assess subjects’ communication abilities, especially involving the communicative use of language in a social context (e.g., “I find it easy to ‘read between the lines’ when someone is talking to me,” or “I am often the last to understand the point of a joke”). The AQ-Comm scale ranges from 0 to 10, with higher scores indicating stronger presence of a pragmatic deficit, a trait associated with autism spectrum disorder (Baron-Cohen, 1988). The subscale has strong discriminative validity, yielding scores that differ significantly between individuals with autism and typically developing individuals (Broadbent et al., 2013).

### EEG data collection

**Fig. 1:**
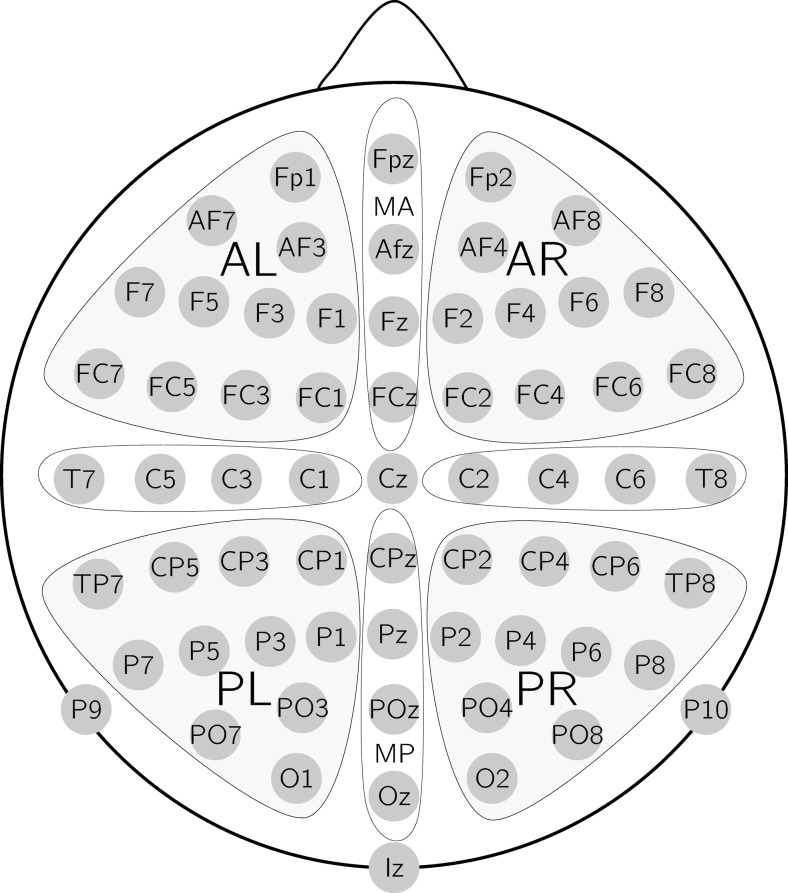
Electrode configuration and the region of interest (ROI) clusters used for statistical analyses. AL = anterior left, AR = anterior right, PL = posterior left, PR = posterior right, MA = midline anterior, MP = midline posterior

The EEG was recorded at a sampling rate of 512 Hz using a BioSemi ActiveTwo system (http://www.biosemi.com) with 64 EEG electrodes in an International 10–20 electrode configuration (see Fig. 1), two additional mastoid electrodes, and four EOG electrodes (left and right horizontal cantus and above/below the right eye), referenced to the common mode sense (CMS; active electrode) and grounded to a passive electrode. The system’s hardware is completely DC-coupled and applies digital low-pass filtering through its ADC’s decimation filter (the hardware bandwidth limit). This has a fifth-order sync response with a 3 dB point at one-fifth of the sample rate (i.e., approximating a low-pass filter at 100 Hz). The EEG was rereferenced to the average of the left and right mastoid electrode offline and filtered (0.1–20 Hz bandwidth filter with 12-dB slope plus 50-Hz Notch filter). The data were then segmented into epochs that started 500 ms before critical word onset and lasted 1,000 ms after word onset and corrected for ocular artefacts using the Grattons and Coles method implemented in BrainVision Analyzer (Brain Products). All epochs were normalized to a 200-ms prestimulus baseline and then semiautomatically screened for artefacts. Seven participants were excluded because of excessive artefacts. Cut-off was 21 artefact-free epochs within each experimental condition, which equals a trial loss of <30 %. For the remaining 23 participants,^2^ individual average ERPs were computed over artefact-free trials (average trial loss 11.7 %) for critical words of each condition.

### Statistical analysis

Using average amplitude per condition across electrodes, 2 (truth-value: true, false) × 2 (mood: counterfactual, hypothetical) repeated-measures ANOVA were performed in the time window of 350–500 ms, which corresponds to the N400-effect. Scalp distributions of the effects were examined using electrode grouping into regions of interest (ROIs; see Fig. 1). Separate analyses were performed for lateral ROIs (AL, AR, PL, PR) using a 2 (truth) × 2 (mood) × 2 (hemisphere: left, right) × 2 (anteriority: anterior, posterior) ANOVA and midline ROIs (MA, MP) using a 2 (truth) × 2 (mood) × 2 (anteriority: anterior, posterior) ANOVA.

## Results

### N400-effects of linguistic mood and real-world truth-value

**Fig. 2:**
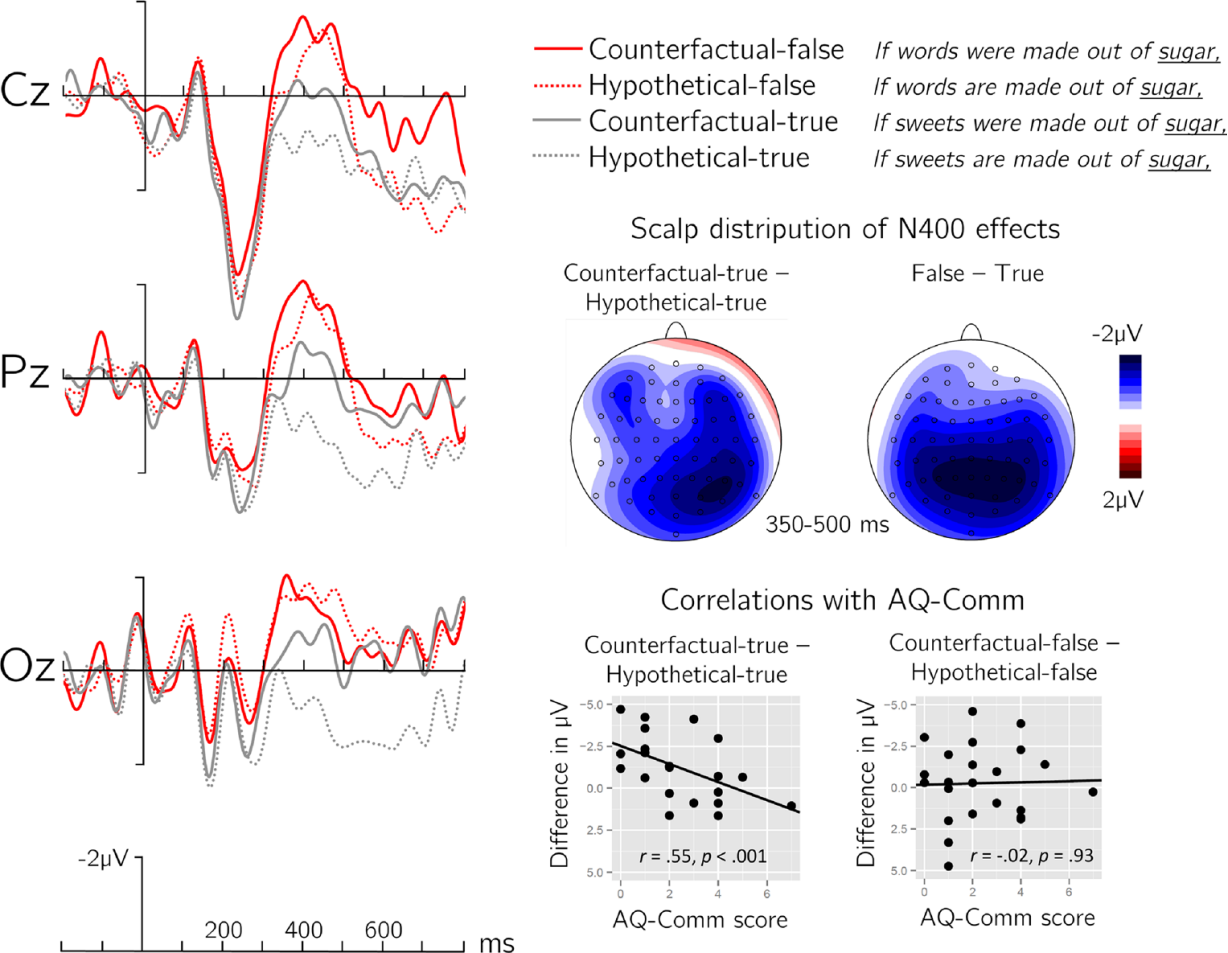
ERP results of counterfactual and hypothetical clauses. Left side: Grand average waveforms timelocked to critical word onset for each of the four experimental conditions. Right side middle: Scalp distributions of the mean difference for counterfactual-true minus hypothetical-true (i.e., the mood difference in true conditions) and for false minus true (i.e., the main effect of truth-value). Right side bottom: Correlation of the Autism Questionnaire Communication score (AQ-Comm) with the voltage difference between true conditions (left) and between false conditions (right) elicited over right hemisphere electrodes. (Color figure online)

Critical words that rendered an antecedent factually false elicited larger (more negative) N400s compared to words rendering an antecedent true (see Fig. 2; a figure showing all electrodes is provided in the Supplementary Material). This was supported by a main effect of truth-value, mean difference 1.16 μV; *F*(1, 22) = 14.10, *p* < .001, η_p_^2^ = .39, in the lateral ROI ANOVA. Furthermore, counterfactual antecedents elicited larger N400s than hypothetical antecedents, reflected in a main effect of mood, mean difference 0.72 μV; *F*(1, 22) = 8.44, *p* < .01, η_p_^2^ = .28. Critically, however, we also observed a robust truth by mood by hemisphere interaction, *F*(1, 22) = 13.42, *p* < .001, η_p_^2^ = .39. Based on the prediction that mood effects should be restricted to true clauses, we resolved this interaction by truth-value, which has the additional advantage of comparing critical words that are equally semantically related to the preceding context. This revealed a significant mood by hemisphere interaction in the true, *F*(1, 22) = 6.81, *p* < .05, and a weaker interaction effect in the false condition, *F*(1, 22) = 4.39, *p* < .05. Further resolving both interaction effects showed that true counterfactual clauses elicited significantly larger N400s compared to true hypothetical clauses in the right, mean difference 1.24 μV; *t*(22) = 3.15, *p* < .01, but not the left hemisphere, mean difference 0.70 μV; *t*(22) = 1.89, *p* = .07. In false conditions, counterfactual and hypothetical clauses did neither differ in the right, mean difference 0.26 μV; *t*(22) < 1, nor in the left hemisphere, mean difference 0.68 μV; *t*(22) = 1.42, *p* = .17. Finally, all false sentences elicited stronger N400s than did true sentences in posterior, mean difference 1.57 μV; *t*(22) = 4.39, *p* < .001, compared to anterior electrodes, mean difference 0.75 μV; *t*(22) = 2.28, *p* < .05, resulting in a significant truth by anteriority interaction, *F*(1, 22) = 7.46, *p* < .05.

The midline repeated-measures ANOVA revealed similar effects. Words that rendered clauses false elicited stronger N400s compared to true continuations, mean difference 1.18 μV; *F*(1, 22) = 13.43, *p* < .001. Counterfactual clauses elicited stronger N400s compared to hypothetical clauses, mean difference 0.72 μV*; F*(1, 22) = 5.16, *p* < .05. The N400s elicited by false clauses were stronger in posterior, mean difference 1.65 μV; *t*(22) = 4.30, *p* < .001, compared to anterior electrodes, mean difference 0.72 μV; *t*(22) = 1.99, *p* = .06, indicated by a truth by anteriority interaction, *F*(1, 22) = 6.36, *p* < .05.

### AQ-Comm correlation analysis

For this analysis, we first calculated an individual N400-effect score as the difference between the amplitudes of counterfactual-true and hypothetical-true conditions in the right hemisphere where this effect was most pronounced. This difference constitutes a score for the individual sensitivity to the pragmatic violation of counterfactuality, with more negative values indicating stronger sensitivity. A correlation analysis showed that individual pragmatic proficiency (AQ-Comm score; *M* = 2.35, *SD* = 1.76; range 0–7) was significantly correlated with the individual size of the pragmatic N400-effect, *r*(21) =.55, *p* < .001, 95 % CIs [.20, .81]. This indicates that pragmatically skilled subjects (with low AQ-Comm scores) showed stronger pragmatic N400 effects compared to subjects with low pragmatic skills (high AQ-Comm scores). No such effect was observed for the difference between mood in the factually false conditions, *r*(21) = -.03, *p* = .93.

### Declarative control sentences

Analyses of the declarative control sentences revealed that words that rendered clauses false elicited larger N400s compared to words that rendered clauses true (mean difference 1.82 μV). A 2 (truth-value) × 2 (anteriority) × 2 (hemisphere) repeated-measures ANOVA showed the corresponding main effect of truth-value, *F*(1, 22) = 15.89, *p* < .001. A similar effect of truth-value was present in the midline ANOVA, mean difference 2.42 μV; *F*(1, 22) = 17.74, *p* < .001.

**Fig. 3:**
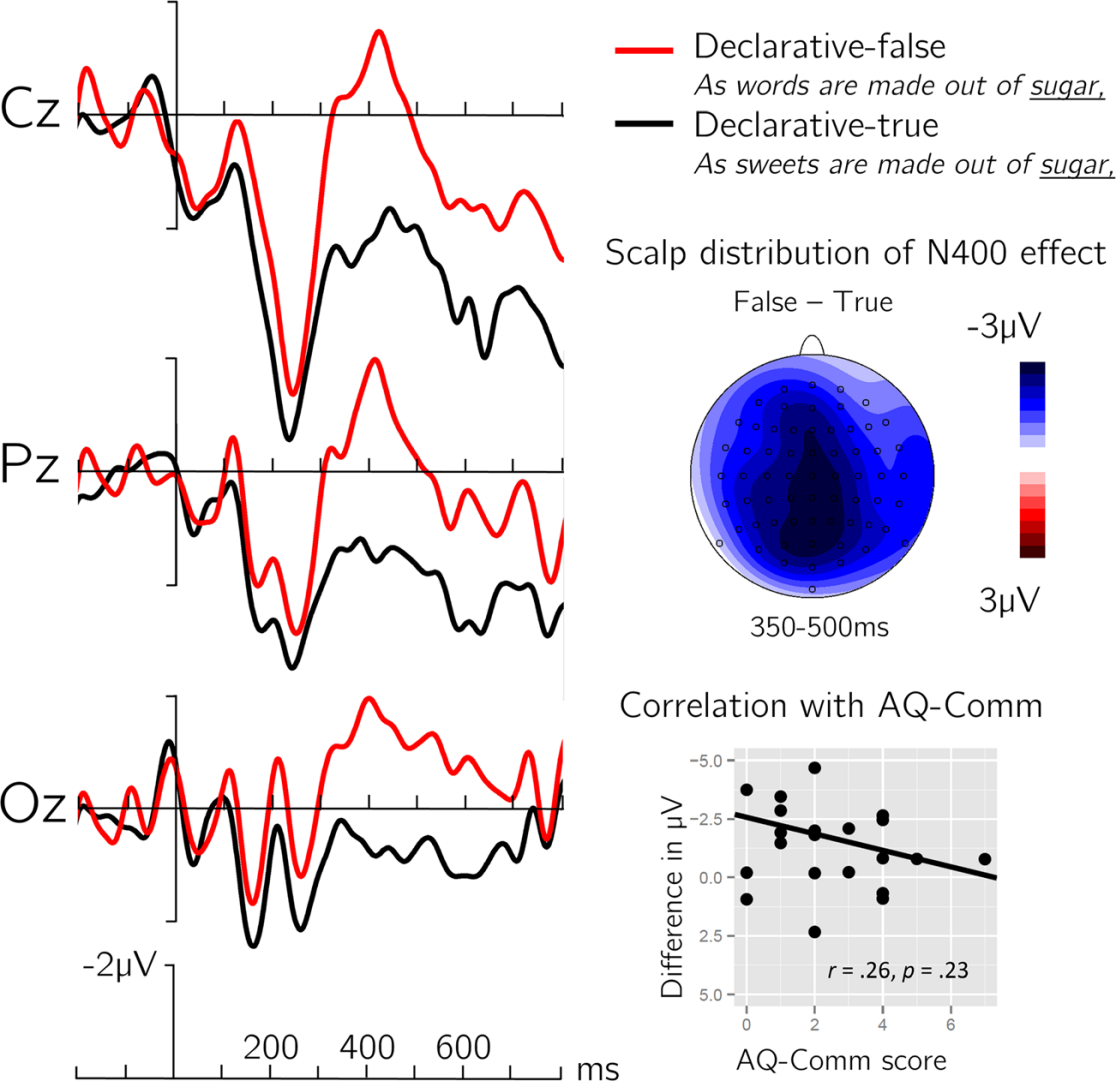
ERP results of declarative control clauses. Left side: Grand average waveforms time-locked to critical word onset. Right side middle: Scalp distributions of the mean difference effect of truth-value. Right side bottom: Correlation of the Autism Questionnaire Communication score (AQ-Comm) with the voltage difference between true and false conditions over right hemisphere electrodes. (Color figure online)

The semantic N400-effect was not significantly correlated with individual AQ-Comm score in the right hemisphere, *r*(21) = .26, *p* = .23, although the slope of the correlation was in the same direction as the statistically significant correlation between the pragmatic N400-effect and the AQ-Comm score (see Fig. 3). A direct comparison of the correlation coefficients showed a marginal but not significant difference, *t*_diff_(20) = 1.15, *p* = .13 (Chen & Popovich, 2002), suggesting that the association between pragmatic skills and the pragmatic N400 effect cannot be confidently concluded to be larger than the association between pragmatic skills and the effect of world knowledge in declarative clauses. The similar direction and magnitude of both correlations suggests the possibility that the individual N400 mood-effect was (partly) driven by a general sensitivity to world knowledge violations. To test this possibility, we computed the partial correlation between AQ-Comm and the pragmatic N400-effect while controlling for individual variation of the semantic N400-effect. The correlation remained stable and statistically significant, *r*(20) = .54, *p* < .01, 95 % CIs [.22, .75], which suggests that the relationship between pragmatic skills and the online effect of counterfactuality cannot be accounted for by a general sensitivity to world knowledge violations.

## Discussion

The present EEG study investigated online comprehension of counterfactual antecedents and the role of pragmatic skills therein. This yielded two novel findings. First, we observed that a subtle difference in linguistic mood (*If sweets were/are made out of sugar*, with subjunctive and indicative mood for counterfactuals and hypotheticals, respectively) was associated with a robust N400 ERP difference. The critical word *sugar* elicited larger N400s following the counterfactual *were* compared to the hypothetical *are*. We take this result to reflect the lower expectancy for the word *sugar* in the counterfactual antecedent compared to in the hypothetical antecedent. Second, this pragmatic-counterfactual N400-effect increased with the pragmatic abilities of the participant. This suggests that pragmatic ability—the knowledge of the communicative and social use of language and successful application of this knowledge in daily life—is associated with better online use of the counterfactual-pragmatic cue conveyed by subjunctive mood. Thus, we take our results to reflect the real-time pragmatic processes involved in creating counterfactual worlds.

In addition to these key findings, we observed larger N400s overall when the critical word had little semantic relationship to the context and conveyed information that was false with respect to world knowledge (*If words were/are made out of sugar*). In these sentences, the ERP responses are primarily driven by lack of contextual facilitation. Absence of a linguistic mood effect here is in line with previous studies that reported absence of a negation effect in sentences such as *A robin is/is not a tree* (e.g., Fischler, Bloom, Childers, Roucos, & Perry, 1983). If negation or counterfactuality does not increase the expectation of the critical word, little effect of truth-value is observed on the N400 (see Nieuwland, 2015, 2016, for a discussion). This is because the N400 does not index the plausibility of the overall proposition, nor does it reflect online verification processes directly, but the extent to which semantic retrieval for incoming words is facilitated by representations of the context, whether the context is counterfactual, hypothetical, or factual.

### Individual differences in online pragmatic comprehension

Our results provide novel experimental evidence for a link between social cognition and the online processing of counterfactual language. Recent results show that people can use their pragmatic knowledge of what constitutes a contextually relevant utterance in a rapid and incremental manner (e.g., Ferguson & Cane, 2015; Nieuwland et al., 2010; Nieuwland & Kuperberg, 2008). This pragmatic knowledge can be used along with the overt language input to generate coarse-grained expectancies about what upcoming information is likely to be encountered. However, individuals may differ greatly in their ability to generate such pragmatic expectancies (Ferguson et al., 2015; Nieuwland et al., 2010). A pattern somewhat similar to the current findings was observed by Nieuwland et al. (2010), namely that pragmatically infelicitous scalar quantifier sentences, such as *Some people have lungs,* elicited larger N400s than felicitous sentences, such as *Some people have pets,* only in individuals with relatively good pragmatic skills (low AQ-Comm scores). Related findings have also been reported for individual differences in empathy, the ability to recognize and experience the emotions of others (Ferguson et al., 2015; Van den Brink et al., 2012). For example, high-empathy individuals showed greater N400-effects of speaker-message incongruence (an adult saying “I cannot sleep without my teddy” compared to a child saying this; Van den Brink et al., 2012).

Findings from the empathy quotient and autism questionnaire may be quite similar. Scores on these questionnaires are strongly correlated and are both employed in the diagnosis of autism spectrum disorder, perhaps because both tap into different aspects of misunderstanding other’s intentions (Baron-Cohen & Wheelwright, 2004; Baron-Cohen et al., 2001; Wheelwright et al., 2006). The inclusion of such measures is reflecting a growing interest to incorporate individual differences in online language processing. It also highlights the increasing acceptance of the view that nonlinguistic processes such as pragmatic inferencing, social stereotyping, or belief computation play an important role during online language processing (Ferguson et al., 2015; Van den Brink et al., 2012). Our results directly support this development by contributing evidence that pragmatic skills play a role in online counterfactual sentence comprehension.

### Counterfactual language and autism

Our results have potential implications for the understanding of counterfactual reasoning deficits in autism. Participants with autism are known to pay less attention to contextual cues during reasoning (De Martino, Harrison, Knafo, Bird, & Dolan, 2008). For example, autists make less use of their world knowledge in conditional reasoning tasks. This lowers their susceptibility to counterexamples to conditional relations, whereas control participants perform less “logical” because of plausibility and reality bias (McKenzie et al., 2010; Pijnacker, Hagoort, Buitelaar, Teunisse, & Geurts, 2009). Autists also have difficulties using discourse or sentence context to disambiguate semantically ambiguous words (F. Happé, 1997). These results have been attributed to weak central coherence—the deficient employment of world knowledge during language understanding (F. G. Happé & Frith, 2006). Such deficit might also be related to autists’ counterfactual reasoning difficulties (Scott et al., 1999).

It should be emphasized that the present study was carried out with healthy students who showed only a subclinical variation of a subcategory of traits associated with autism. However, our results indicate that autism-like traits have a graded impact on basic semantic processing during language comprehension. This finding itself seems consistent with a central coherence explanation, namely that weaker pragmatic skills are associated with less use of world knowledge to predict upcoming information. However, the observation that the pragmatic correlation could not be explained by participants’ sensitivity to world knowledge violations does not support this conclusion. Instead, our results suggest that self-reported pragmatic skills are predictive of using pragmatic information in an online manner during comprehension, which is qualitatively different form a general impairment to recruit world knowledge.

Our results are thus in line with the assumption that it is the pragmatic difficulty to create a possible world what underlies autists’ counterfactual reasoning impairments. Because understanding other people’s communicative intentions is required to successfully incorporate pragmatic cues during counterfactual language comprehension, autists might have particular difficulties at this early point of counterfactual interpretation. Less sensitive to the explicit cue of counterfactuality, they are more likely to miss the communicative intention of counterfactuals’ pretence and subsequently revert to shallow reasoning strategies (Leevers & Harris, 2000).

However, further research is required to clarify the exact relation between social-communicative abilities and counterfactual reasoning and to overcome some of the limits of the current study. The design of our study did not allow us to directly investigate the relationship between the processing of counterfactual antecedents and consequents. One important question for further research is how counterfactual antecedent comprehension affects the processing of subsequent consequents as well as conditional reasoning performance in general. In particular, individuals with lower pragmatic skills might still be able to establish counterfactual meaning, but this processing step might be delayed. Another open question is whether the current result replicate in counterfactual antecedents with highly predictable critical words that are semantically related to the context (e.g., *If WWII had been won by theNazis,* or *If sweets were not made out ofsugar*). Our results may also be fairly specific to English, where verb-mood is available before the critical word. This can work differently in other languages (e.g., German; see Kulakova et al., 2014), and studying counterfactual constructions in different languages can therefore clarify the time course of incremental pragmatic processing as a function of the order of incoming information. Further investigations are also required to clarify whether the current online results indeed are best explained by pragmatic skills, as perhaps the most important caveat to our conclusions is that we did not investigate the possible role of other individual differences. For instance, grammatical skills might play an important role in identifying the counterfactual meaning of subjunctive mood in the antecedent, given that linguistic mood is a grammatical feature. Children with autism who generally have problems with counterfactuals usually do not have a grammatical impairment (Weismer et al., 2011), suggesting that grammatical deficits are not necessary to disrupt counterfactual comprehension, but grammatical deficits (possibly independently of pragmatic skills) might indeed be sufficient to disrupt counterfactual comprehension. Finally, a validation of our findings with a clinically autistic sample could further help to identify how more severe pragmatic deficits affect online counterfactual sentence processing.

## Conclusion

Counterfactual “what-if” thought enables people to go beyond the here and now and into hypothetical worlds they know to be false. The present study examined the brain’s electrophysiological correlates of creating such possible worlds during online language comprehension and investigated whether social-communicative pragmatic skills predicted the online use of counterfactual cues. Our results show that counterfactuality, as conveyed by subjunctive mood, is quickly incorporated during language comprehension and leads comprehenders to decrease the expectations about upcoming information they would normally base on real-world knowledge. Words that are true with respect to this knowledge therefore incur a semantic processing cost, as reflected in larger N400 amplitude, in counterfactuals compared to hypotheticals (*If sweets were/are made of sugar*). However, individuals who are better at understanding the communicative intentions of other people are more likely to reduce knowledge-based expectations in counterfactuals. These results are the first demonstration of the real-time pragmatic processes involved in creating possible worlds.

## Electronic supplementary material

Below is the link to the electronic supplementary material.

ESM 1 (PDF 601 kb)

Supplemental Fig. 1 (GIF 2.21 mb)

High Resolution (TIF 9.51 mb)

## Footnotes

1 The labels *true* and *false* are not meant to suggest that counterfactual or hypothetical antecedents themselves (*If words were/are made out of sugar,*…) have a clear truth-value. Rather, following a logical-philosophical tradition (e.g., Lewis 1973), antecedent truth-value labels are derived from the truth-values of corresponding declarative propositions (*Words are made out of sugar*).

2 We decided against additional data collection based on a power analysis (G*Power; Faul, Erdfelder, Lang, & Buchner, 2007) establishing the required sample size to detect a significant strong correlation effect (*r* = .50) between individual pragmatic skills and N400 amplitude based on comparable previous findings (Ferguson et al., 2015; Nieuwland et al., 2010). With the recommended settings of alpha = .05 and power = .80 (Cohen, 1992), a sample size of *N* = 23 is sufficient to detect the expected effect.
