# Supplementary material for: Pragmatic skills predict online counterfactual comprehension: Evidence from the N400
Source: Cogn Affect Behav Neurosci. 2016 May 9;16(5):814–24. doi: 10.3758/s13415-016-0433-4 (PMC5018041; doi:10.3758/s13415-016-0433-4)
Supplement: Supplementary file 1 — (PDF 601 kb) [file 13415_2016_433_MOESM1_ESM.pdf]

Complete stimulus list

1. If/As dogs/fish are/were born with two gills, Terriers/salmon can/could easily survive in water without ever breaching.
2. If/As buses/airplanes are/were equipped with two wings, coaches/jets can/could transport people by flying over long distances.
3. If/As beer/jam is/was made from fresh berries, Guinness/marmalade can/could be the colour red or even purple.
4. If/As cups/snowballs are/were made out of snow, teacups/snowmen can/could be damaged by sunshine or by heat.
5. If/As dogs/rabbits are/were fed on fresh vegetables, Huskies/bunnies can/could be fed with carrots and white cabbage.
6. If/As beer/vinegar is/was used to make salads, ale/balsamic can/could be used as dressing for rocket salad.
7. If/As cats/experts are/were able to give advice, Siamese/specialists can/could easily serve as consultants or professional mentors.
8. If/As birds/humans are/were able to understand language, gulls/children can/could make some silly jokes just for fun.
9. If/As rodents/writers are/were known to like literature, hamsters/poets can/could enjoy a short poem by William Blake.
10. If/As worms/grasshoppers are/were born with long legs, grubs/locusts can/could move around by jumping in the air.
11. If/As sport/smoking is/was known to promote diseases, yoga/cigarettes can/could eventually make people sick and very miserable.
12. If/As cocoa/gunpowder is/was used to make explosives, chocolate/TNT can/could be part of grenades and of bombs.
13. If/As birds/aristocrats are/were able to build houses, eagles/earls can/could live in huge mansions in the countryside.
14. If/As jewels/vegetables are/were used to prepare soup, diamonds/onions can/could be used in minestrone or onion soup.
15. If/As cheese/a-bombs are/were made with added uranium, Cheddar/detonations can/could make many people die in an instant.
16. If/As aliens/celebrities are/were living somewhere around Hollywood, Martians/Madonna can/could own houses in Pasadena or Los Angeles.
17. If/As condiments/drugs are/were used for occasional recreation, garlic/alcohol can/could be consumed to relax together with friends.
18. If/As bone/paper is/was made out of wood, skeletons/sheets can/could very easily be lit with a match.

19. If/As crocodiles/elephants are/were born with a trunk, alligators/tuskers can/could reach food from trees with high branches.
20. If/As fish/spiders are/were able to make webs, goldfish/harvestmen can/could catch food in midair without any problems.
21. If/As inches/clocks are/were used to measure time, rulers/watches can/could easily show the hour and minute precisely.
22. If/As reptiles/humans are/were able to make cars, lizards/men can/could get a nice cabriolet to impress girls.
23. If/As nuts/humans are/were known to have feelings, peanuts/people can/could sometimes be very sad or feel happy.
24. If/As insects/mammals are/were able to give milk, flies/sows can/could soothe offspring by breast-feeding it with care.
25. If/As houses/pills are/were the size of marbles, villas/painkillers can/could very easily be swallowed by small children.
26. If/As ducks/marble is/was as heavy as stone, drakes/marbles can/could never move over water without sinking immediately.
27. If/As noodles/sweets are/were made from pure sugar, spaghetti/lollipops can/could bring about serious toothache when eaten frequently.
28. If/As tigers/mice are/were afraid of big cats, cougars/rats can/could be scared by Siamese cats and flee.
29. If/As corn/mushrooms are/were known to contain toxins, popcorn/toadstools can/could be used for poisoning a disliked enemy.
30. If/As rodents/mosquitoes are/were known to drink blood, hamsters/gnats can/could feed on the wounds of various animals.
31. If/As women/men are/were able to grow beards, girls/guys can/could have a pretty goatee or nice sideburns.
32. If/As vitamins/psychedelics are/were consumed to promote hallucinations, Carotene/LSD can/could easily bring about disorientation or unpleasant paranoia.
33. If/As bread/fuel is/was made out of petrol, buns/spills can/could cause very severe pollution of the environment.
34. If/As pots/clothes are/were made out of wool, cups/jumpers can/could sometimes by accident shrink when boil washed.
35. If/As sociology/mathematics is/was the science of numbers, sociologists/mathematicians can/could easily solve difficult equations just for fun.
36. If/As weapons/water is/was useful for fighting fire, pistols/groundwater can/could be used by firefighters during an operation.
37. If/As soap/water is/was used for making tea, shampoo/tap-water can/could be used for brewing Earl Grey tea.

38. If/As pebble/wheat is/was used for making bread, stones/flour can/could be found in buns and in loafs.
39. If/As daylight/darkness is/was indicative of the night, sunrises/sunsets can/could occur in the evening but not morning.
40. If/As poker/soccer is/was played with a ball, gamblers/strikers can/could be injured by balls hitting their heads.
41. If/As educators/sailors are/were usually working in ships, teachers/seamen can/could easily get very seasick during their work.
42. If/As cats/dinosaurs are/were known to be extinct, Persians/T-rex can/could be studied by palaeontologists with great interest.
43. If/As motorbikes/boats are/were used for crossing water, mopeds/canoes can/could serve to cross lakes or large rivers.
44. If/As calculators/cameras are/were used to produce photos, abacuses/polaroids can/could serve to take portraits or panorama photos.
45. If/As cars/drugs are/were sold in the pharmacy, Fords/aspirin can/could be bought at Boot's without any problems.
46. If/As vegetables/flutes are/were used for making music, cucumbers/piccolos can/could be used in orchestras or in bands.
47. If/As fruit/pencils are/were used for making drawings, bananas/crayons can/could be used to sketch a nice landscape.
48. If/As students/zombies are/were keen on eating brains, undergrads/undead can/could eat pounds of neurons at lunch break.
49. If/As electro-shocks/yoga is/was known to induce relaxation, stun-belts/exercises can/could make people more calm and rather peaceful.
50. If/As coffee/sleep is/was useful to get rest, espresso/sleeping can/could make people very relaxed and very calm.
51. If/As sausages/nectars are/were made out of fruit, bangers/juice can/could sometimes taste like apple or like orange.
52. If/As mechanics/barbers are/were specialised in doing haircuts, machinists/hairdressers can/could quickly provide a fringe or a perm.
53. If/As Christians/Muslims are/were known to worship Allah, Protestants/Islamists can/could pray in the mosque during the day.
54. If/As membranes/frontiers are/were known to separate countries, cell-walls/borders can/could be relevant to politicians and the military.
55. If/As magicians/soldiers are/were known to use weapons, wizards/officers can/could easily handle a machinegun or a rifle.
56. If/As sandals/snowshoes are/were worn in the snow, flip-flops/moonboots can/could be appropriate for winter and cold weather.

57. If/As blood/plants are/were known to contain chlorophyll, flesh/grass can/could be the colour green on most occasions.
58. If/As factories/bees are/were known to produce honey, manufacturers/beehives can/could be plundered by bears which are hungry.
59. If/As aubergines/cocaine is/was known to be illegal, greengrocers/dealers can/could be put in prison by the police.
60. If/As water/caries is/was harmful to the teeth, tap-water/sugar can/could eventually make teeth decay and ache terribly.
61. If/As applause/matches are/were used to make fire, clapping/strikes can/could instantly produce a blaze which would spread.
62. If/As bugs/horses are/were somewhat bigger than humans, earwigs/ponies can/could be used for riding and for transportation.
63. If/As America/Cuba is/was known for being communist, Washington/Havana can/could be opposed to capitalism at any level.
64. If/As biscuits/cigarettes are/were made out of tobacco, cookies/Marlboro's can/could cause very strong addiction if consumed frequently.
65. If/As squaws/mermaids are/were able to breathe underwater, Pocahontas/Arielle can/could live in the ocean without any problems.
66. If/As sound/light is/was perceived with the eyes, music/paintings can/could be appreciated by vision but not hearing.
67. If/As dogs/cows are/were known to like grass, greyhounds/calves can/could be fed with hay or with straw.
68. If/As boats/rockets are/were known to travel space, ships/shuttles can/could transport people to galaxies very far away.
69. If/As mania/depression is/was a state of sadness, maniacs/depressed can/could very rarely feel happy or pleasantly excited.
70. If/As wars/monasteries are/were known to be peaceful, soldiers/monks can/could be considered as pacifistic and calm people.
71. If/As homeopathy/pharmaceutics is/was treated as a science, globuli/painkillers can/could be classified as medicine without any doubt.
72. If/As coffins/vegetables are/were sold at the supermarket, caskets/salad can/could be bought at Tesco's or in Sainsbury's.
73. If/As jars/photos are/were used to preserve memories, jam/albums can/could have many different topics such as 'Holidays'.
74. If/As drivers/pilots are/were able to operate planes, racers/aviators can/could easily steer big jets carrying many passengers.
75. If/As phobics/knights are/were known to be brave, phobia/knighthood can/could be regarded as virtuous and earn respect.

76. If/As prostitution/carpentry is/was known as a craft, prostitutes/carpenters can/could start their own company without legal problems.
77. If/As anorexia/obesity is/was associated with being fat, anorexics/obese can/could only fit in large jumpers and trousers.
78. If/As Frenchmen/Englishmen are/were used to talking English, Parisians/Londoners can/could all easily understand Americans or even Australians.
79. If/As fruit/rice is/was edible only after boiling, apples/risotto can/could only be eaten cooked but not raw.
80. If/As insects/athletes are/were used to frequent exercising, ants/runners can/could aim for a medal at the Olympics.
81. If/As stones/clouds are/were made out of water, pebbles/rain can/could easily make people wet in the spring.
82. If/As shops/churches are/were a place for prayer, bakeries/cathedrals can/could be visited for worship on Sunday mornings.
83. If/As clouds/music is/was made out of notes, rain/songs can/could have very beautiful melodies to sing along.
84. If/As tombs/roller-coasters are/were known to be fun, graveyards/fairs can/could be places offering entertainment to many people.
85. If/As words/sweets are/were made out of sugar, sentences/candy can/could make people very fat when consumed frequently.
86. If/As drugs/spices are/were used to prepare food, aspirin/curry can/could be used as seasoning for hot dishes.
87. If/As waste/gold is/was known to be valuable, trash/rings can/could sometimes be very expensive and sometimes unaffordable.
88. If/As snow/flames are/were known to be hot, ice/fire can/could be used for warming your hands on.
89. If/As pastry/sushi is/was prepared with fresh fish, croissants/temaki can/could be filled with salmon or raw tuna.
90. If/As fights/elections are/were used to establish governments, brutality/popularity can/could make a successful politician come to power.
